# Serum antibody titers against *Porphyromonas gingivalis* are associated with oral and gut microbiota

**DOI:** 10.3389/froh.2026.1956003

**Published:** 2026-09-15

**Authors:** Yoichiro Nakajima, Nahoko Kato-Kogoe, Takako Yasuda, Shoichi Sakaguchi, Kuniyasu Kamiya, Junya Inubushi, Wataru Hamada, Minori Horiuchi, Asako Kudo, Kouji Tsuda, Takashi Nakano, Junko Tamaki, Takaaki Ueno

**Affiliations:** 1 Department of Dentistry and Oral Surgery, Faculty of Medicine, Osaka Medical and Pharmaceutical University, Takatsuki, Japan; 2 Research and Development Department, Sunstar Inc., Takatsuki, Japan; 3 Department of Microbiology and Infection Control, Faculty of Medicine, Osaka Medical and Pharmaceutical University, Takatsuki, Japan; 4 Basic Medical Sciences Region, Kobe City College of Nursing, Kobe, Japan; 5 Department of Hygiene and Public Health, Faculty of Medicine, Osaka Medical and Pharmaceutical University, Takatsuki, Japan

**Keywords:** gut microbiota, oral microbiota, periodontitis, *Porphyromonas gingivalis*, serum antibody

## Abstract

**Introduction:**

*Porphyromonas gingivalis* (*P. gingivalis*) is a keystone pathogen in the oral microbiota and has attracted attention for its association with periodontal as well as systemic diseases. While animal studies have suggested that *P. gingivalis* affects the gut microbiota and may trigger related pathologies, evidence from human studies remains insufficient. In this study, we aimed to identify characteristics of the oral and gut microbiota associated with serum antibody titers against *P. gingivalis*, which serve as an indicator of *P. gingivalis* infection.

**Methods:**

Serum antibody titers against *P. gingivalis* were measured in 149 community-dwelling older adults, who were classified by their quartile into high (PgAb_H), medium (PgAb_M), and low (PgAb_L). Simultaneously collected saliva and stool samples were subjected to 16S rRNA metagenomic analysis to compare the bacterial composition of the oral and gut microbiota between the PgAb_H and PgAb_L groups. Using Linear Discriminant Analysis Effect Size (LEfSe), we identified the bacteria whose abundance differed significantly between the groups. The prevalence of bacterial genera commonly present in both the oral and gut microbiota of the same individual was compared between the groups.

**Results:**

Significant differences were observed between the PgAb_H and PgAb_L groups regarding the bacterial composition of the oral microbiota (unweighted UniFrac distance, *p* = 0.001; weighted UniFrac distance, *p* = 0.041) and that of the gut microbiota (weighted UniFrac distance, *p* = 0.045). LEfSe analysis identified several bacterial genera in the oral and gut microbiota whose abundances differed between the two groups. Although the two groups did not differ in the number of bacterial genera shared between the oral and gut microbiota in the same individual, the proportion of individuals possessing *Haemophilus* in both the oral and gut microbiota was significantly lower in the PgAb_H group than in the PgAb_L group (*p* = 0.003).

**Conclusion:**

This study characterized the oral and gut microbiota profiles associated with serum antibody titers against *P. gingivalis*. These findings deepen our understanding of how *P. gingivalis* infection affects the human body beyond the oral cavity, and suggest that serum antibody titers against *P. gingivalis* could serve as a potentially meaningful indicator for health management.

## 1 Introduction

More than 700 species of bacteria inhabiting the oral cavity constitute the oral microbiota, which plays a significant role in health and disease (1, 2). Recent evidence suggests that the oral microbiota influences the gut microbiota, and that their interaction contributes to systemic diseases (3–5). Under certain conditions, oral bacteria and their metabolic byproducts can disrupt the balance of the gut microbiota via the bloodstream or the gastrointestinal tract, potentially contributing to the pathogenesis of various diseases, including chronic inflammatory conditions, metabolic disorders, neurological diseases, and cancer (6, 7). Among the various oral bacteria, *Porphyromonas gingivalis* (*P. gingivalis*) has attracted particular attention, since accumulating evidence suggests that it can translocate to the gut, alter the balance of the gut microbiota, and cause both local and systemic effects (3, 4, 8).

*P. gingivalis* is a major periodontal pathogen. Since it disrupts the balance of the oral microbiota and causes inflammation, it is recognized as a keystone pathogen in the onset and progression of periodontitis (9, 10). Periodontal disease, which involves bacterial infections and inflammation, affects not only oral health but also systemic health (11, 12). Recent research suggests that an imbalance in the oral and gut microbiota in periodontal disease may contribute to the worsening of inflammatory and metabolic diseases (13). In mice, the salivary microbiota of patients with periodontitis disrupts the balance of the gut microbiota, affecting various diseases such as intestinal diseases (14), systemic lipid metabolism (15), rheumatoid arthritis (16) and Alzheimer's disease (17). Recent clinical studies in humans have also reported that patients with periodontitis have a distinctive gut microbiota (18), and that this changes following periodontal treatment (19, 20).

In addition to the oral cavity, *P. gingivalis* has also been detected at lesion sites of several extraoral diseases*, including* atherosclerotic plaques, joints of patients with rheumatoid arthritis, brain tissue of patients with Alzheimer's disease, and mucosal tissue of patients with gastrointestinal cancer (21, 22). Studies over several years have demonstrated that *P. gingivalis* infection is closely associated with the onset and progression of these systemic diseases, and have elucidated the underlying mechanisms. Among these mechanisms, disruption of the gut microbiota has been proposed as a key pathway linking *P. gingivalis* infection to systemic disease pathology, and at least two non-mutually exclusive routes: direct translocation of oral bacteria to the gut via swallowing (14, 23–26), and indirect effects of periodontitis- and *P. gingivalis* infection- associated systemic inflammation on the gut epithelial barrier and mucosal immune system (12, 25). Animal studies have shown that oral administration of *P. gingivalis* alters the gut microbiota and impairs the intestinal barrier function, thereby inducing inflammation in the gut and throughout the body (23–26). However, evidence of the association between *P. gingivalis* infection and the gut microbiota in humans remains insufficient.

*P. gingivalis* infection causes an increase in host serum IgG antibody levels against the bacterium (27, 28). *P. gingivalis* antibodies likely reflect current and past exposure to *P. gingivalis,* and the host systemic immune response to it*.* Notably, serum antibody levels are determined primarily by carriage of the pathogen rather than by the clinical presence of periodontitis itself (27); because periodontitis is a categorical diagnosis based on a threshold of clinically detectable tissue destruction, whereas persistent *P. gingivalis* carriage continuously drives humoral immune activation, antibody titers more directly track cumulative exposure history than a cross-sectional clinical diagnosis. Moreover, unlike clinical periodontal parameters such as probing pocket depth and clinical attachment level, which can fluctuate substantially depending on treatment history and oral hygiene at the time of examination, serum antibody titers are less susceptible to such short-term fluctuation (29), owing to the long half-life of circulating IgG and the persistence of immunological memory (30). Serum *P. gingivalis* antibody titers may therefore serve as a more stable indicator of the cumulative, longer-term history of infection and exposure than clinical parameters reflecting periodontal status at a single time point. Elevated serum antibody titers against *P. gingivalis* have been reported in patients with periodontitis (31–33) and other related systemic diseases, such as cardiovascular diseases (34, 35), autoimmune diseases (36), neurological diseases (37), and gastrointestinal diseases (38). Therefore, serum antibody titers against *P. gingivalis*, which enable quantitative and objective assessment of *P. gingivalis* infection through a blood test, may be clinically useful for screening *P. gingivalis* infection associated with these chronic systemic inflammatory diseases.

*P. gingivalis* infection has been shown to affect the gut microbiota in association with periodontal disease and abnormalities in the oral microbiota, in animal studies; however, studies in humans remain insufficient. We hypothesized that individuals with higher serum *P. gingivalis* antibody titers, reflecting a greater cumulative burden of *P. gingivalis* infection and exposure, would have experienced more persistent colonization and proliferation of this keystone pathogen, resulting in more pronounced alterations in the composition of the oral microbiota, and that these oral changes would in turn be associated with alterations in the gut microbiota through both direct translocation of oral bacteria and indirect, inflammation-mediated pathways. Here, we aimed to evaluate the association between serum *P. gingivalis* antibody titers and the oral and gut microbiota in humans, and to characterize bacterial genera shared between the oral and gut microbiota of the same individuals, which may reflect colonization of the gut by translocated oral bacteria (6), as indirect evidence of oral-to-gut bacterial transfer, as previously reported in an elderly population (39). To address this, we simultaneously collected blood, saliva, and stool samples from the same individuals, and characterized the oral and gut microbiota associated with serum antibody titers against *P. gingivalis* in community-dwelling older individuals in Japan.

## 2 Materials and methods

### 2.1 Participants

This study was conducted in accordance with the Declaration of Helsinki and its latest revisions and was approved by the Ethics Committee of Osaka Medical and Pharmaceutical University, Takatsuki, Japan (Approval No. 2145). Written informed consent was obtained from all participants.

The participants in this study were 149 individuals selected from residents who took part in the “Takatsuki Study” between May and November 2018. The Takatsuki Study was a prospective cohort study of residents aged ≥ 65 years in Takatsuki City designed to investigate the relationship between oral health and various systemic diseases. Inclusion criteria were the availability of the samples and systemic and oral information required for this study. Exclusion criteria were current smoking or antibiotic use, based on self-report, and edentulism, based on oral examination.

### 2.2 Measurement of serum antibody titers against *P. gingivalis*

Serum antibody titers against *P. gingivalis* were measured as described previously (40). In brief, a chemiluminescent enzyme immunoassay (CLEIA) was performed using a flow-through membrane and an automatic chemiluminescence immunoassay analyzer (Toyobo, Osaka, Japan) in accordance with the manufacturer's instructions (35). The arginine gingipain (Rgp) sequence of *P. gingivalis* was targeted as the antigen (32). The antibody titer (U/mL) was calculated from the calibration curve of the positive serum. The participants were divided into three groups, namely high (PgAb_H), medium (PgAb_M), and low (PgAb_L), based on the quartiles of their serum antibody titers against *P. gingivalis*. To evaluate the association between serum antibody titers against *P. gingivalis* and the oral and gut microbiota, we compared the oral and gut microbiota between the PgAb_H and PgAb_L groups. Comparative analyses including the PgAb_M group were also performed; however, no consistent trend was observed for this group relative to the PgAb_H and PgAb_L groups. Therefore, to more clearly evaluate the influence of the degree of serum *P. gingivalis* antibody titer, we focused our comparison on the PgAb_H and PgAb_L groups.

### 2.3 Oral examination

A full-mouth clinical oral examination was performed and periodontal conditions were assessed by measuring periodontal pocket depth (PPD) and bleeding on probing (BOP) at six sites on each tooth, as described previously (40). We calculated the periodontal inflamed surface area (PISA), a periodontal parameter used to quantify the extent of inflamed periodontal tissue (41). The presence and severity of periodontitis were classified as periodontal health, mild, moderate, or severe, based on the case definitions for population-based surveillance of periodontitis (42).

### 2.4 Oral and gut sample collection

Saliva samples for oral bacterial analysis and stool samples for gut bacterial analysis were collected from the same participants at the same period. Samples were collected according to previously reported methods (43). In short, saliva samples were collected in the morning after an overnight fast using the SalivaBio® Oral Swab and Swab Storage Tube (Salimetrics, State College, Pennsylvania, USA) (44). Stool samples were collected by the participants themselves using the Mykinso® Stool Collection Kit (Cykinso, Tokyo) and mailed to the research center.

### 2.5 DNA extraction, 16S rRNA sequencing, and taxonomic classification

To analyze the microbial communities in the saliva and stool samples, we performed DNA extraction, 16S rRNA sequencing, and taxonomic classification according to previously reported methods (40). To put it simply, after homogenizing the samples, we extracted DNA using GENE PREP STAR PI-480 (Kurashiki Spinning Co., Ltd., Osaka) in accordance with the manufacturer's instructions. The V1-V2 region of the 16S rRNA gene was amplified by PCR, a library was prepared, and 250-bp paired-end sequences were generated using the MiSeq Reagent Kit v2 (Illumina, San Diego, CA, USA). An average of 31,081 non-chimeric sequence reads were denoised and quality-filtered using DADA2 within the Quantitative Insights into Microbial Ecology 2 (QIIME2) version 2025.4. The minimum depth cutoff for rarefaction was set at 10,000 and each amplicon sequence variant (ASV) was assigned to the curated Greengenes2 2024.09 full-length reference database using a naive Bayes classifier.

### 2.6 Quantification of periodontal bacteria in saliva sample

The DNA extracted from saliva samples, as described above, was quantified for total bacterial count and the counts of the periodontal bacteria *P. gingivalis*, *Tannerella forsythia* (*T. forsythia*), and *Treponema denticola* (*T. denticola*) using Genopearl® (Mitsubishi Chemical Corporation, Japan).

### 2.7 Statistical analysis

Comparisons of subject characteristics between groups were evaluated using the Mann–Whitney *U* test or the Kruskal–Wallis test for quantitative data and the chi-square test for categorical data, as appropriate, followed by Bonferroni correction for multiple comparisons. IBM SPSS Version 27 was used for data analysis, and the level of statistical significance was set at *p* < 0.05.

Intra-group *α* diversity of the microbial community was assessed using the observed features (ASVs) index and the Shannon index, and comparisons between groups were made using the Kruskal–Wallis test. *β*-diversity among groups was assessed using phylogeny-based indices (unweighted and weighted UniFrac distances) and comparisons were made using permutational multivariate analysis of variance (PERMANOVA). Principal coordinates analysis (PCoA) was used to visualize UniFrac distances, and the first two principal-coordinate axes were displayed. PERMANOVA was performed on the full distance matrix using 999 permutations. In the PCoA plots, 95% data ellipses were drawn for each group assuming a bivariate normal distribution (radius = √(2·F₀.₉₅(2, n−1)) in units of the sample standard deviation, equivalent to stat_ellipse(type = “norm”) in ggplot2), and group centroids were indicated by diamonds. The QIIME2 software was used for the analyses. The Linear Discriminant Analysis Effect Size (LEfSe) algorithm was used to detect bacteria whose abundances differed across the groups. The alpha parameter for LEfSe's pairwise tests was set to 0.05, and the log score threshold was set to 2.0.

## 3 Results

### 3.1 Characteristics of the participants

The median (interquartile range) serum antibody titer against *P. gingivalis* in the 149 participants was 1,129.2 (250.7–4,104.5) (Supplementary Figure 1). Table 1 shows the baseline characteristics of the three groups, namely PgAb_H (*n* = 38), PgAb_M (*n* = 73), and PgAb_L (*n* = 38), classified according to the quartiles of serum antibody titers against *P. gingivalis*. The three groups differed significantly in age, sex, and number of teeth, while no significant differences were observed in BMI, or prevalence of hypertension, diabetes, and dyslipidemia. Although age and sex did not differ significantly between the PgAb_H and PgAb_L groups, participants in the PgAb_H group had significantly fewer teeth than those in the PgAb_L group (*p* = 0.018).

**Table 1:** Baseline characteristics of the study population (*n* = 149).

Variables	Total	PgAb_L	PgAb_M	PgAb_H		PgAb_L vs. PgAb_H
	*n* = 149	*n* = 38	*n* *=* 73	*n* = 38	*p*-value	*p*-value
Age (years)	75 (71–78)	75 (72–78)	74 (71–76)	77 (74–82)	0.001	0.062
Sex [Female, *n* (%)]	98 (65.8%)	26 (68.4%)	54 (74.0%)	18 (47.4%)	0.018	0.189
BMI (kg/m^2^)	22.4 (20.5–24.3)	21.6 (20.2–23.6)	22.4 (20.6–24.1)	23.1 (21.2–24.6)	0.263	0.283
Hypertension [*n* (%)]	80 (53.7%)	21 (55.3%)	35 (47.9%)	24 (63.2%)	0.305	1.000
Diabetes [*n* (%)]	26 (17.4%)	4 (10.5%)	14 (19.2%)	8 (21.1%)	0.415	0.722
Dyslipidemia [*n* (%)]	51 (34.2%)	13 (34.2%)	28 (38.4%)	10 (26.3%)	0.447	1.000
Number of teeth	25 (21–28)	27 (23–28)	25 (21–28)	24 (17–27)	0.023	0.018

Results are expressed as the median (interquartile range) or *n* (number).

*p*-values are calculated using the Kruskal–Wallis test for continuous variables and the chi-square test for categorical variables. For PgAb_L vs. PgAb_H, *p*-values are the results of comparisons using the Mann–Whitney *U* test or chi-square test, followed by Bonferroni correction.

Hypertension was defined as a blood pressure > 140/90 mmHg or the use of antihypertensive drugs.

Diabetes was defined as HbA1c > 6.5% (NGSP) or use of oral antidiabetic drugs or insulin therapy.

Dyslipidemia was defined as LDL-C > 140 mg/dL, HDL-C < 40 mg/dL, and triglycerides > 150 mg/dL or the use of anti-dyslipidemic drugs.

### 3.2 Periodontal status

The status of periodontal disease differed significantly across the three groups (*p* = 0.021). In the PgAb_H group, the PISA, number of mobility teeth, and the number of teeth with a periodontal pocket depth ≥ 6 mm were significantly higher than in the PgAb_L group (*p* = 0.006, *p* = 0.045, and *p* = 0.013, respectively, Figure 1A). The findings confirmed the association between high *P. gingivalis* antibody titers and severity of periodontal disease, consistent with previous studies.

**Figure 1:**
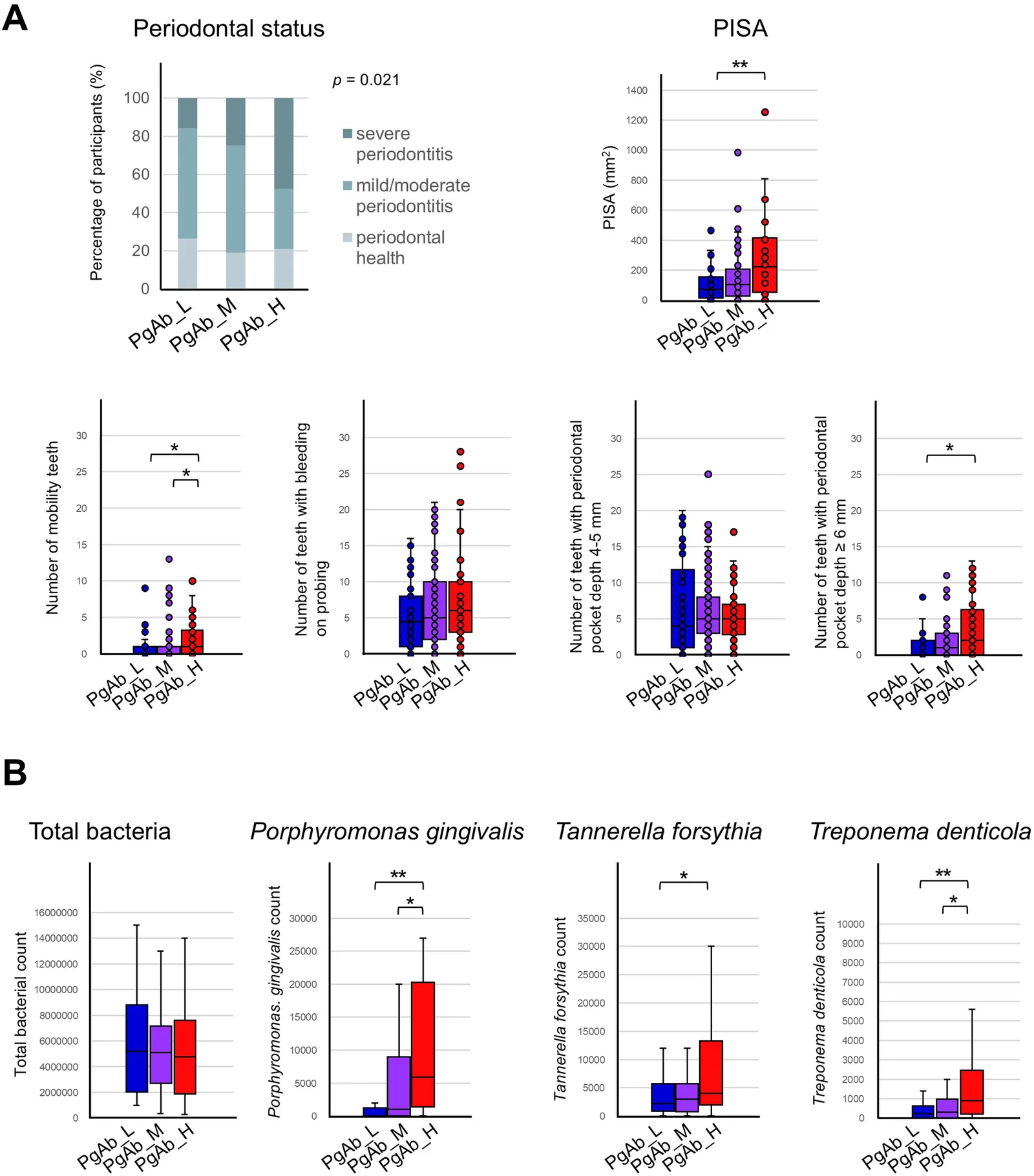
Periodontal status and periodontal bacteria in saliva. **(A)** The status of periodontal disease, periodontal inflamed surface area (PISA), the number of mobility teeth, the number of teeth with bleeding on probing, the number of teeth with a periodontal pocket depth of 4–5 mm, and the number of teeth with a periodontal pocket depth ≥ 6 mm in the PgAb_L, PgAb_M and PgAb_H groups are shown. **(B)** The total bacterial count and the bacterial counts of *Porphyromonas gingivalis*, *Tannerella forsythia*, and *Treponema denticola* in the PgAb_L, PgAb_M, and PgAb_H groups are shown. * *p* < 0.05, ** *p* < 0.01. Kruskal–Wallis test followed by Wilcoxon rank-sum test with Bonferroni correction for quantitative data; chi-square test for categorical data.

### 3.3 Periodontal bacteria in saliva

The mean total bacterial count in saliva samples was 7.2 × 10^6^/*μ*l in the PgAb_L group, 6.3 × 10^6^/μl in the PgAb_M group, and 5.5 × 10^6^/μl in the PgAb_H group, with no significant difference across the groups (Figure 1B). The detection rate of *P. gingivalis* in saliva was 26.3% in the PgAb_L group, 54.8% in the PgAb_M group, and 81.6% in the PgAb_H group. Detection rates of the other major periodontal bacteria, *T. forsythia* (79.0%, 83.6%, and 94.7% in the PgAb_L, PgAb_M, and PgAb_H groups, respectively) and *T. denticola* (52.6%, 54.8%, and 81.6% in the PgAb_L, PgAb_M, and PgAb_H groups, respectively), were similarly higher in the PgAb_H group than in the PgAb_L group. The counts of *P. gingivalis*, *T. forsythia*, and *T. denticola* were significantly higher in the PgAb_H group than in the PgAb_L group (Figure 1B). The results suggested that individuals with higher *P. gingivalis* antibody titers are more likely to have periodontal bacteria detected, and in higher counts, in their saliva.

### 3.4 Oral microbiota

The oral microbiota was classified into 330 genera, 146 families, 79 orders, 32 classes, and 21 phyla. The proportions of the 20 most abundant genera were 86.0% and 87.8% in the PgAb_H and PgAb_L groups, respectively (Figure 2A). PgAb_H contained 177 genera, 43 of which were not detected in PgAb_L, whereas PgAb_L contained 180 genera, 46 of which were not detected in PgAb_H. A total of 72 genera were common to both groups in at least 30% of the participants; in addition to these, 15 genera were detected in the PgAb_H group, and 3 were detected in the PgAb_L group (Figure 2A).

**Figure 2:**
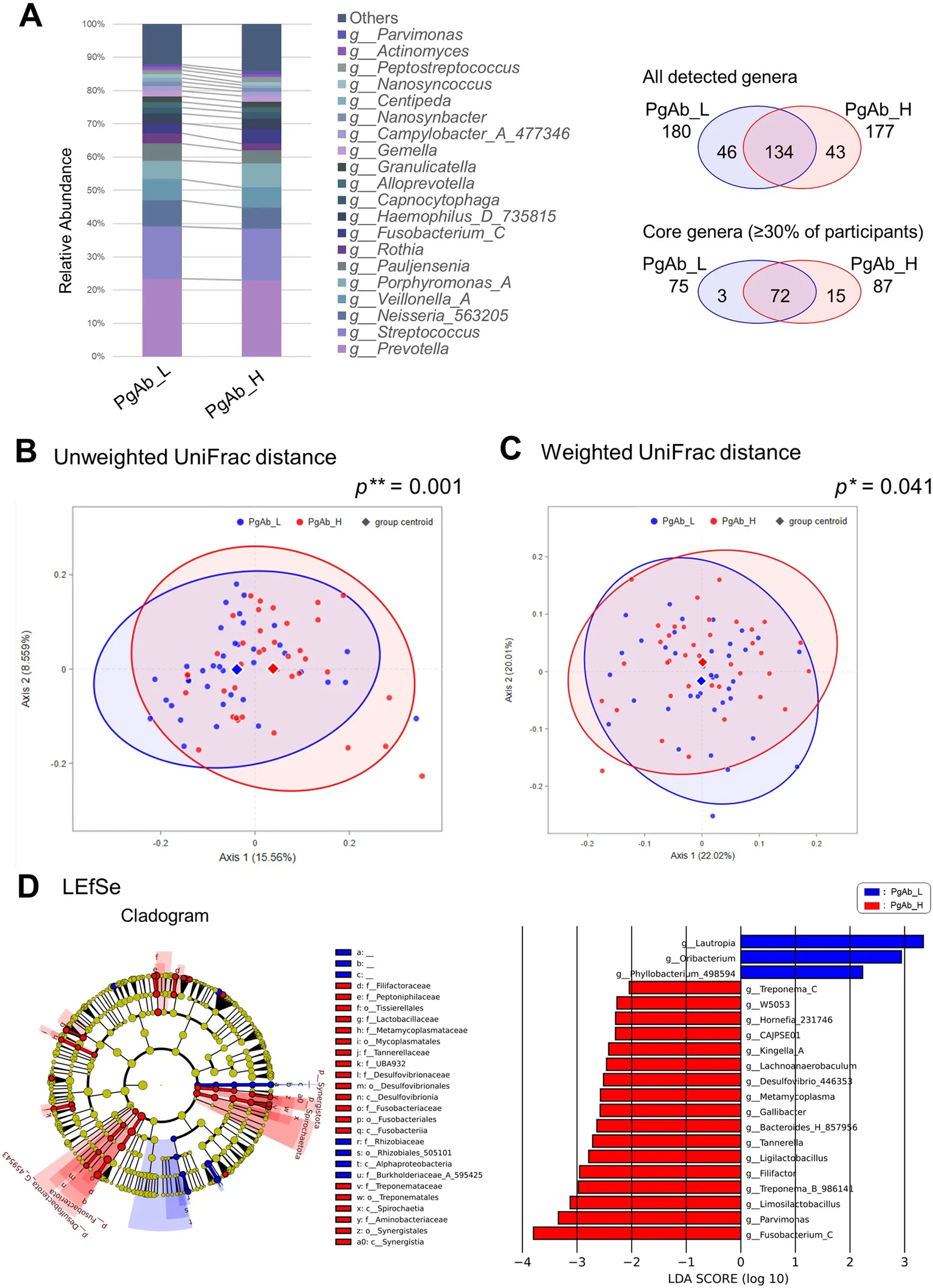
Differences in oral microbiota between groups. **(A)** Taxonomic composition of the oral microbiota at the genus level (top 20 genera) in the PgAb_H (*n* = 38) and PgAb_L (*n* = 38) groups, shown as a bar plot. Venn diagrams showing the number of bacterial genera detected in the oral microbiota of the PgAb_H and PgAb_L groups. The upper diagram includes all genera detected in at least one participant; the lower diagram includes only genera detected in at least 30% of participants. **(B,C)** *β*-diversity of the gut microbiota based on unweighted **(B)** and weighted **(C)** UniFrac distances, visualized using the first two axes of principal coordinates analysis (PCoA). Ellipses indicate 95% data ellipses, and diamonds indicate group centroids in the two-dimensional ordination. The PgAb_H (*n* = 38) and PgAb_L (*n* = 38) groups were compared by PERMANOVA with 999 permutations; *, *p* < 0.05; **, *p* < 0.01. **(D)** Bacterial taxa with significantly different abundance between the PgAb_H and PgAb_L groups, identified by Linear Discriminant Analysis Effect Size (LEfSe). The cladogram illustrates these taxa across taxonomic levels, with each ring representing a different rank (phylum to genus). The histogram shows the corresponding LDA scores (threshold ≥ 2.0) for each taxon enriched in the PgAb_H (red) or PgAb_L (blue) group.

To evaluate the association between serum antibody titers against *P. gingivalis* and the oral microbiota, we compared the oral microbiota between the PgAb_H and PgAb_L groups. Analysis of *α*-diversity revealed no significant difference in species richness (Observed features, *p* = 0.412) or evenness (Shannon index, *p* = 0.280) between the two groups. Beta diversity analysis revealed significant differences in bacterial composition between the two groups based on both unweighted (PERMANOVA, 999 permutations, *p* = 0.001; Figure 2B) and weighted (PERMANOVA, 999 permutations, *p* = 0.041; Figure 2C) UniFrac distances.

Specific bacteria that differed in relative abundance from the phylum to genus levels between the PgAb_H and PgAb_L groups were identified using LEfSe analysis. At the genus level, the PgAb_H group had higher abundances than the PgAb_L group for genera such as *Fusobacterium* and *Parvimonas*, and lower abundances for genera such as *Lautropia* and *Oribacterium* (Figure 2D). The results revealed that serum antibody titers against *P. gingivalis* were associated with the abundance of specific bacteria in the oral microbiota.

### 3.5 Gut microbiota

The gut microbiota was classified into 549 genera, 141 families, 60 orders, 27 classes, and 18 phyla. The proportions of the 20 most abundant genera were 65.3% and 68.8% in the PgAb_H and PgAb_L groups, respectively (Figure 3A). PgAb_H included 362 genera, 71 of which were not detected in PgAb_L while PgAb_L included 336 genera, 45 of which were not detected in PgAb_H. Among more than 30% of the participants, 90 genera were detected in both groups, in addition to the 17 genera detected in PgAb_H and 4 detected in PgAb_L (Figure 3A).

**Figure 3:**
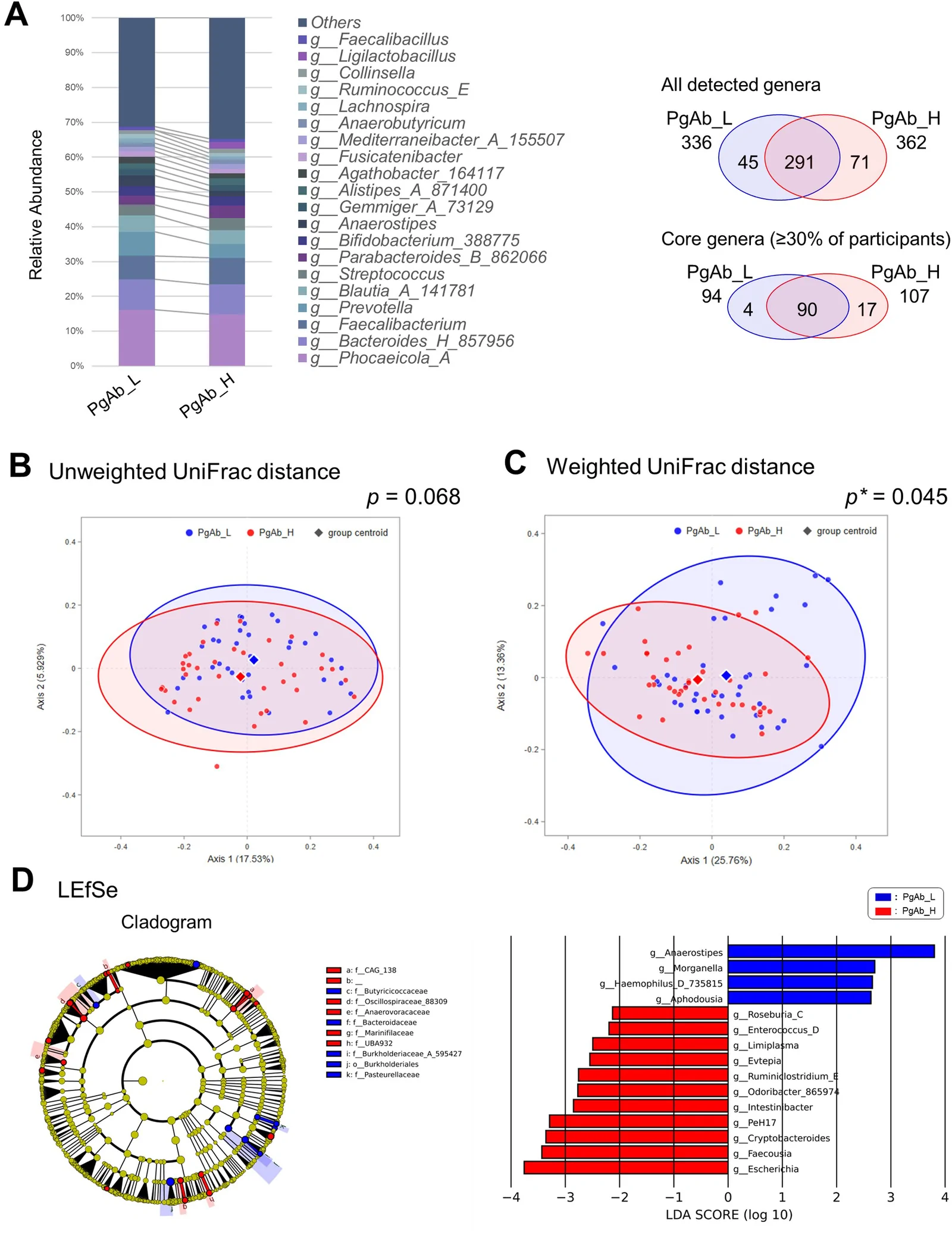
Differences in gut microbiota between groups. **(A)** Taxonomic composition of the gut microbiota at the genus level (top 20 genera) in the PgAb_H (*n* = 38) and PgAb_L (*n* = 38) groups, shown as a bar plot. Venn diagrams showing the number of bacterial genera detected in the gut microbiota of the PgAb_H and PgAb_L groups. The upper diagram includes all genera detected in at least one participant; the lower diagram includes only genera detected in at least 30% of participants. **(B,C)** *β*-diversity of the gut microbiota based on unweighted **(B)** and weighted **(C)** UniFrac distances, visualized using the first two axes of principal coordinates analysis (PCoA). Ellipses indicate 95% data ellipses, and diamonds indicate group centroids in the two-dimensional ordination. The PgAb_H (*n* = 38) and PgAb_L (*n* = 38) groups were compared by PERMANOVA with 999 permutations; *, *p* < 0.05. **(D)** Bacterial taxa with significantly different abundance between the PgAb_H and PgAb_L groups, identified by Linear Discriminant Analysis Effect Size (LEfSe). The cladogram illustrates these taxa across taxonomic levels, with each ring representing a different rank (phylum to genus). The histogram shows the corresponding LDA scores (threshold ≥ 2.0) for each taxon enriched in the PgAb_H (red) or PgAb_L (blue) group.

To evaluate the association between serum antibody titers against *P. gingivalis* and the gut microbiota, we compared the gut microbiota of the PgAb_H and PgAb_L groups. In the analysis of *α*-diversity, no significant difference was observed between the two groups in terms of species richness (observed features, *p* = 0.278) or evenness (Shannon index, *p* = 0.461). In beta diversity analysis, the unweighted UniFrac distance was not significant (PERMANOVA, 999 permutations, *p* = 0.068; Figure 3B), whereas the weighted UniFrac distance was (PERMANOVA, 999 permutations, *p* = 0.045; Figure 3C), indicating a difference in bacterial composition between the two groups.

The LEfSe analysis showed that, at the genus level, the PgAb_H group exhibited higher abundance than the PgAb_L group in genera such as *Escherichia*, *Faecousia*, and *Cryptobacteroides*, and lower abundance in genera such as *Anaerostipes*, *Morganella*, and *Haemophilus* (Figure 3D). Therefore, serum antibody titers against *P. gingivalis* were suggested to be associated with the abundance of specific bacteria in the gut microbiota.

### 3.6 Common bacterial genera shared in the oral and gut microbiota

To evaluate whether *P. gingivalis* antibody titers were associated with the relationship between the oral and gut microbiota, we compared the common bacterial genera shared in the oral and gut microbiota between the PgAb_H and PgAb_L groups.

The bacterial genera detected in at least one participant were 223 in the oral microbiota and 407 in the gut microbiota, of which 105 were detected in both oral and gut microbiota. In the PgAb_L group, 180 genera were identified in the oral microbiota, and 336 were identified in the gut microbiota, of which 87 were detected in both the oral and gut microbiota. In the PgAb_H group, 177 genera were identified in the oral microbiota, 362 were identified in the gut microbiota, and 62 were detected in both oral and gut microbiota. (Figure 4A). The proportion of these shared oral-gut genera relative to the total gut microbiota was slightly lower in the PgAb_H group (17.1%) than in the PgAb_L group (25.9%).

**Figure 4:**
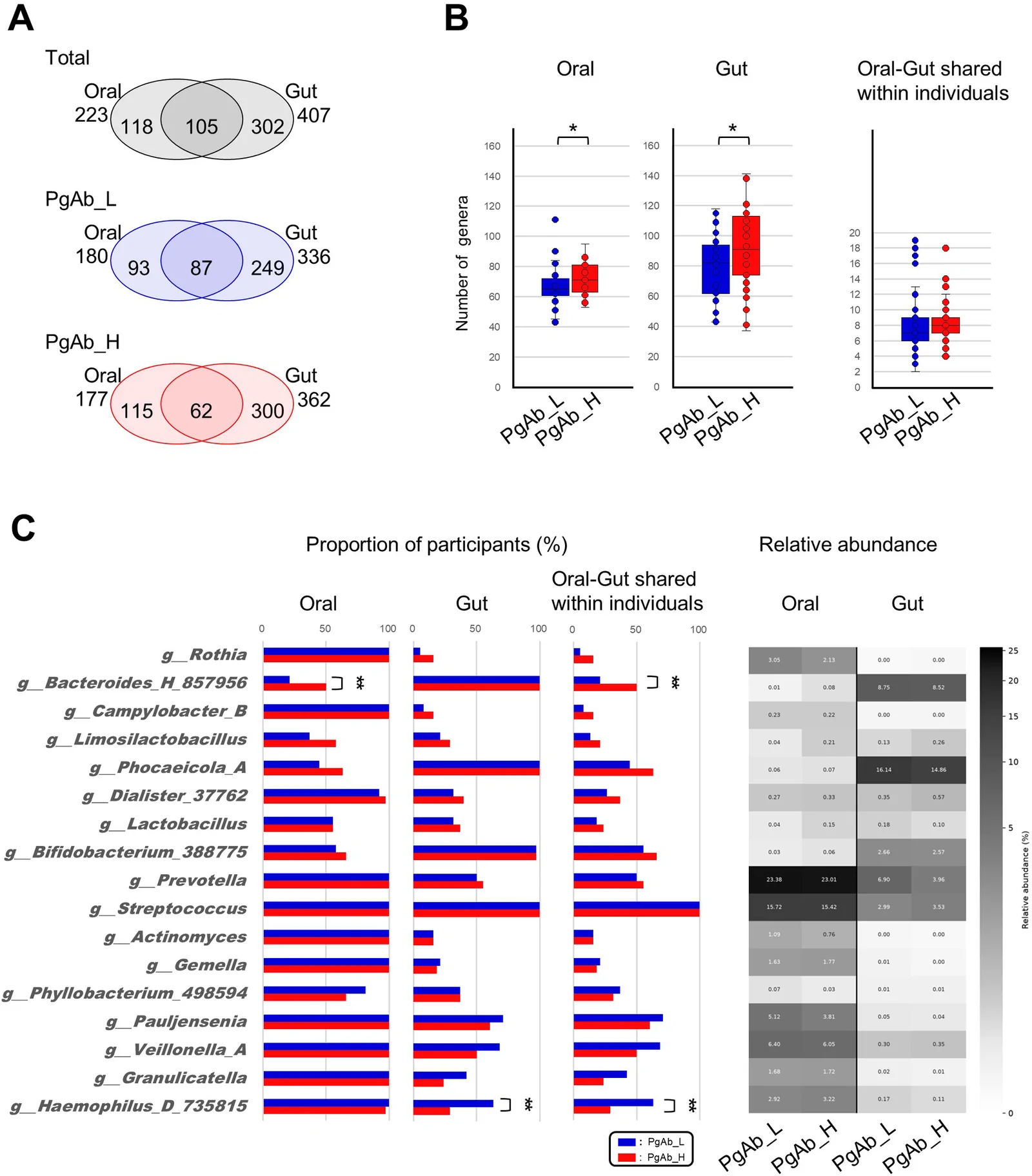
Common bacterial genera shared in the oral and gut microbiota. **(A)** Venn diagrams showing the number of bacterial genera at least one participant detected in the oral and gut microbiota of all participants, the PgAb_H group, and the PgAb_L group. **(B)** The number of bacterial genera detected in the oral microbiota (left) and gut microbiota (middle) of each participant, and the number of genera shared between the oral and gut microbiota within the same individual (right) are shown. * *p* < 0.05, Mann–Whitney *U* test. **(C)** Proportion of participants in the PgAb_H and PgAb_L groups possessing specific bacterial genera, shown separately for the oral microbiota (left), the gut microbiota (middle) and genera shared between the oral and gut microbiota within the same individual (right). ** *p* < 0.01, chi-square test. The heatmap shows the relative abundance of each bacterial genus in the oral and gut microbiota of the two groups.

The number of bacterial genera detected in the oral and gut microbiota of each participant was significantly higher in the PgAb_H group than in the PgAb_L group (*p* = 0.038 in oral and *p* = 0.049 in gut). Among the bacterial genera detected in the gut microbiota, the number of genera also detected in the oral microbiota of the same individuals ranged from 2 to 19, with a median of 8; no significant difference was observed between the PgAb_H and PgAb_L groups (Figure 4B). The proportion of these shared bacteria in each participant's gut microbiota ranged from 3.0% to 44.2% (median: 9.1%), with no significant difference between the groups.

Of the 105 genera detected in the oral and gut microbiota, 70 were shared by at least one individual in both the oral and gut microbiota. The 70 shared bacterial genera included *Bacteroides* and *Limosilactobacillus*, which were shown to be more abundant in PgAb_H than in PgAb_L, *Phyllobacterium*, which was shown to be less abundant in the LEfSe analysis of the oral microbiota (Figure 2D), and *Haemophilus*, which was shown to be less abundant in the LEfSe analysis of the gut microbiota (Figure 3D). The proportions of individuals possessing these 70 shared bacterial genera were determined. *Streptococcus* was detected in the oral and gut microbiota of all participants (100%). *Rothia*, *Bacteroides*, *Campylobacter*, and *Limosilactobacillus* tended to be detected more frequently in the PgAb_H group than in the PgAb_L group. The proportion of individuals harboring *Haemophilus* in both the oral and gut microbiota was significantly lower in the PgAb_H group (28.9%) compared with the PgAb_L group (63.2%) *(p* = 0.003; Figure 4C). There was no significant difference between groups in the relative abundance of these bacterial genera in the oral and gut microbiota (Figure 4C).

## 4 Discussion

The current study revealed that the oral and gut microbiomes of individuals with high serum antibody titers against *P. gingivalis* differ from those of individuals with low titers, and identified specific bacterial genera for which significant differences in abundance and prevalence in oral and gut microbiota were observed. The data suggested that *P. gingivalis* infection may be associated with both oral and gut microbiota in humans, thereby advancing our understanding of the systemic effects of *P. gingivalis* infection and suggesting that health management based on serum antibody titers against *P. gingivalis* may be potentially meaningful.

High antibody titers against *P. gingivalis* indicate that individuals have been strongly exposed to *P. gingivalis* in the past or present, resulting in the activation of a systemic immune response. *P. gingivalis* infection and the associated systemic immune response potentially affect the balance between the oral and gut microbiota. Animal studies have shown that *P. gingivalis* infection plays a central role in disrupting the balance of the oral microbiota, is associated with an imbalance in the gut microbiota, and is linked to systemic diseases (23, 26). However, evidence to validate the findings in humans is lacking. Therefore, to verify the findings in humans, we focused on serum antibody titers against *P. gingivalis* as an indicator of *P. gingivalis* infection.

The serum antibody titers to *P. gingivalis* have been reported to be associated with the clinical symptoms of periodontal disease (33). In this study, compared to the low-*P. gingivalis* antibody-titer groups, the high-titer groups had more severe periodontal disease and higher numbers of periodontal bacteria, including *P. gingivalis,* in their saliva, thereby validating the previous findings. Since *P. gingivalis* antibody titers reflect the status of *P. gingivalis* infection based on the host's immune response to the bacterium, they are considered useful indicators for evaluating the systemic effects of periodontal disease (45). Because periodontal disease is increasingly recognized as a risk factor for various systemic diseases, *P. gingivalis* antibody titers have attracted growing attention (32), as they could enable screening for periodontal disease through a simple blood test, particularly useful in clinical settings without dental specialists. Although *P. gingivalis* plays a central role in the onset and progression of periodontitis, *P. gingivalis* colonization does not necessarily lead to disease, and virulence is expressed only in the context of a polymicrobial community (11, 46). *P. gingivalis* has been reported to modulate the host immune response and cause inflammation; through interactions with other microorganisms, it forms biofilms and disrupts the balance of the oral microbiota (10). Based on these findings, the present study focused on characterizing the oral microbiota associated with *P. gingivalis* infection in humans.

The current study showed that the bacterial composition of the oral microbiota differed between groups with high or low *P. gingivalis* antibody titers. Among the bacterial genera abundantly present in the high-*P. gingivalis* antibody titer-group, many such as *Fusobacterium*, *Parvimonas*, *Treponema*, *Filifactor*, and *Tannerella* are associated with periodontal diseases (20). This could be explained by the fact that, among the participants with high *P. gingivalis* antibody titers, there were many with severe periodontal disease. In particular, within the genus *Fusobacterium*, *Fusobacterium nucleatum* is an oral bacterium that has been extensively studied alongside *P. gingivalis*. Recent reports have described its interactions with *P. gingivalis* within biofilms (47), as well as its relationship with the balance of the oral microbiota (48). Therefore, our findings highlighted the relationship between *P. gingivalis* and *Fusobacterium* in oral microbiota.

In the present study, bacterial composition of the gut microbiota differed between individuals with high *P. gingivalis* antibody titers and those with low titers. This result is interesting, as it suggests that *P. gingivalis* infection may affect the gut microbiota in humans. The effects of *P. gingivalis* infection on gut microbiota have been extensively studied in animal experiments (8, 26). Furthermore, in recent years, the characteristics of the gut microbiota in patients with periodontal disease have become better understood (18, 19, 49). In this study, many subjects with high *P. gingivalis* antibody titers had severe periodontal disease, which may have contributed to changes in their gut microbiota. We identified specific bacterial genera whose abundances differed significantly between the two groups. In particular, the fact that the abundance of *Odoribacter* was higher in individuals with high *P. gingivalis* antibody titers is consistent with reports on the gut microbiota of Japanese patients with periodontal disease (20). The finding that the genus *Escherichia* was more abundant in the gut microbiota of individuals with high *P. gingivalis* antibody titers might indicate an imbalance in the gut microbiota.

Growing evidence suggests that an increase in oral bacteria in the gut microbiota may worsen outcomes in various systemic diseases (5, 6). Therefore, in this study, we evaluated the association between bacterial genera common to both oral and gut microbiota and *P. gingivalis* infection. The results showed no significant difference between individuals with high and low *P. gingivalis* antibody titers regarding the number of bacterial genera shared between the oral and gut microbiota of the same individual. Reports indicate that oral bacteria are frequently found in the gut microbiota of individuals with severe periodontal disease (49, 50). The fact that the periodontal status varied across participants with high *P. gingivalis* antibody titers in this study could be the cause of this result. Additionally, since previous reports indicated the proportion of oral bacteria in the gut microbiota to be higher in older adults (39), our results could be related to the participants being older adults.

The proportion of individuals possessing *Haemophilus* in their gut microbiota was lower in those with high *P. gingivalis* antibodies than in those with low titers. While *Haemophilus* was detected in the oral microbiota of almost all participants. Since the abundance of *Haemophilus* in the gut microbiota increased in patients with IgA deficiency (51), IgA may play an important role in regulating the colonization of *Haemophilus* in the gut. We had previously reported that a strong salivary IgA response to this bacterium might affect the oral microbiota (52). Therefore, individuals with high *P. gingivalis* antibody titers might have had a strong IgA response to this bacterium, which possibly suppressed its colonization in the gut. The role of immune response in microbiota has not yet been fully elucidated, and further analysis to clarify its function would be beneficial.

The study had some limitations. First, since the data in this study were cross-sectional, speculation on the causal relationship between *P. gingivalis* infection and the oral and gut microbiota was not possible. Therefore, the next important step was to confirm the association in interventional studies. Second, we did not perform any tests to assess *P. gingivalis* infection outside the oral cavity. Serum *P. gingivalis* antibody titers have been reported to be elevated not only in periodontitis but also in association with various systemic diseases, including cardiovascular disease, autoimmune disease, neurological disease, and gastrointestinal disease; therefore, it cannot be determined whether the elevated antibody titers observed in this study were attributable to oral infection, extraoral infection/exposure, or both. Identifying the specific site of infection underlying the elevated antibody titers remains a subject for future research. Third, all participants were elderly Japanese residents of Takatsuki City. Therefore, the results of this study might not be generalizable to other populations. Since the participants’ backgrounds, such as age, ethnicity, and dietary culture, influence the oral and gut microbiota, evaluations in various settings would be necessary. Fourth, the 16S metagenomic analysis used in this study evaluated the relative abundance of bacteria rather than their absolute abundance, and focused on species composition and community diversity. To obtain additional information on the functions of specific bacteria or microbial communities, it would be necessary to include more detailed data such as long-read sequencing and various omics analyses. In addition, the LEfSe analysis used to identify differentially abundant taxa was not accompanied by a strict correction for multiple testing (e.g., false discovery rate correction); therefore, findings concerning genera with relatively low abundance should be considered exploratory and will require validation in future studies. Nevertheless, the current study is a preliminary pilot project, and larger-scale studies would be required to confirm the observations and thoroughly investigate the causal relationship between *P. gingivalis* infection and the oral and gut microbiota.

## 5 Conclusion

We characterized the oral and gut microbiota associated with serum antibody titers against *P. gingivalis.* These findings provide strong evidence for a significant association between elevated *P. gingivalis* antibody titers and alterations in the gut microbiota, supporting the oral-gut axis hypothesis and establishing a basis for future interventional studies to confirm causality.

## Data Availability

The datasets presented in this study can be found in online repositories. The names of the repository/repositories and accession number(s) can be found below: https://www.ddbj.nig.ac.jp/, SAMD01942434-SAMD01942581.

## References

[1] Tian S Ding T Li H. Oral microbiome in human health and diseases. mLife. (2024) 3(3):367–83. 10.1002/mlf2.12136 39359681 PMC11442140

[2] Baker JL Mark Welch JL Kauffman KM McLean JS He X. The oral microbiome: diversity, biogeography and human health. Nat Rev Microbiol. (2024) 22(2):89–104. 10.1038/s41579-023-00963-6 37700024 PMC11084736

[3] Tan X Wang Y Gong T. The interplay between oral microbiota, gut microbiota and systematic diseases. J Oral Microbiol. (2023) 15(1):2213112. 10.1080/20002297.2023.2213112 37200866 PMC10187086

[4] Lu Y Li Z Peng X. Regulatory effects of oral microbe on intestinal microbiota and the illness. Front Cell Infect Microbiol. (2023) 13:1093967. 10.3389/fcimb.2023.1093967 36816583 PMC9928999

[5] Kunath BJ De Rudder C Laczny CC Letellier E Wilmes P. The oral-gut microbiome axis in health and disease. Nat Rev Microbiol. (2024) 22(12):791–805. 10.1038/s41579-024-01075-5 39039286

[6] Hernández-Cabanyero C Vonaesch P. Ectopic colonization by oral bacteria as an emerging theme in health and disease. FEMS Microbiol Rev. (2024) 48(2):fuae012. 10.1093/femsre/fuae012 PMC11065354 38650052

[7] Zhu J Jiang Z Yu F Gao L Wang X Wang Q. Integrated oral-gut microbiota therapy: a novel perspective on preventing bacterial translocation for systemic disease management. Front Cell Infect Microbiol. (2025) 15:1641816. 10.3389/fcimb.2025.1641816 40792109 PMC12336206

[8] Olsen I Yamazaki K. Can oral bacteria affect the microbiome of the gut? J Oral Microbiol. (2019) 11(1):1586422. 10.1080/20002297.2019.1586422 30911359 PMC6427756

[9] Lamont RJ Koo H Hajishengallis G. The oral microbiota: dynamic communities and host interactions. Nat Rev Microbiol. (2018) 16(12):745–59. 10.1038/s41579-018-0089-x 30301974 PMC6278837

[10] Curtis MA Garnett JA Darveau RP. The keystone-pathogen hypothesis updated: the role of Porphyromonas gingivalis in periodontitis. J Periodontal Res. (2025) 0:1–19. 10.1111/jre.70050 41174353

[11] Hajishengallis G Lamont RJ Koo H. Oral polymicrobial communities: assembly, function, and impact on diseases. Cell Host Microbe. (2023) 31(4):528–38. 10.1016/j.chom.2023.02.009 36933557 PMC10101935

[12] Hajishengallis G Chavakis T. Local and systemic mechanisms linking periodontal disease and inflammatory comorbidities. Nat Rev Immunol. (2021) 21(7):426–40. 10.1038/s41577-020-00488-6 33510490 PMC7841384

[13] Yamazaki K Kamada N. Exploring the oral-gut linkage: interrelationship between oral and systemic diseases. Mucosal Immunol. (2024) 17(1):147–53. 10.1016/j.mucimm.2023.11.006 38007003 PMC11222583

[14] Qian J Tao Q Shen Y Wang L Wang M Wang N. Periodontitis salivary microbiota exacerbates colitis by CXCL3 derived from gut microbiota-induced macrophages. Microbiome. (2025) 13(1):255. 10.1186/s40168-025-02218-3 41373016 PMC12715969

[15] Bao J Huang M Mao H Yao Y Zhang S Li L. Comparative effects of periodontitis—versus periodontal health-derived saliva on systemic lipid metabolism in mice: mediation through oral-gut axis. J Oral Microbiol. (2026) 18(1):2630494. 10.1080/20002297.2026.2630494 41710692 PMC12912216

[16] Ge R Liu R Zhou Y Zhuang Z Mao H Liao W. Periodontitis salivary Microbiota exacerbates murine rheumatoid arthritis via gut dysbiosis and immune dysregulation. Faseb j. (2025) 39(23):e71282. 10.1096/fj.202502610R 41321261 PMC12666613

[17] Lu J Zhang S Huang Y Qian J Tan B Qian X. Periodontitis-related salivary microbiota aggravates Alzheimer's Disease via gut-brain axis crosstalk. Gut Microbes. (2022) 14(1):2126272. 10.1080/19490976.2022.2126272 36175166 PMC9542625

[18] Lourenço TGB de Oliveira AM Tsute Chen G Colombo APV. Oral-gut bacterial profiles discriminate between periodontal health and diseases. J Periodontal Res. (2022) 57(6):1227–37. 10.1111/jre.13059 36261869

[19] Baima G Ferrocino I Del Lupo V Colonna E Thumbigere-Math V Caviglia GP. Effect of periodontitis and periodontal therapy on oral and gut microbiota. J Dent Res. (2024) 103(4):359–68. 10.1177/00220345231222800 38362600

[20] Miyauchi E Yamazaki K Tsuboi Y Nakajima T Ono S Mizuno K. Patients with periodontitis exhibit persistent dysbiosis of the gut microbiota and distinct serum metabolome. J Oral Microbiol. (2025) 17(1):2499284. 10.1080/20002297.2025.2499284 40351551 PMC12064113

[21] Bregaint S Boyer E Fong SB Meuric V Bonnaure-Mallet M Jolivet-Gougeon A. Porphyromonas gingivalis outside the oral cavity. Odontology. (2022) 110(1):1–19. 10.1007/s10266-021-00647-8 34410562

[22] Dominy SS Lynch C Ermini F Benedyk M Marczyk A Konradi A. Porphyromonas gingivalis in Alzheimer's disease brains: evidence for disease causation and treatment with small-molecule inhibitors. Sci Adv. (2019) 5(1):eaau3333. 10.1126/sciadv.aau3333 30746447 PMC6357742

[23] Arimatsu K Yamada H Miyazawa H Minagawa T Nakajima M Ryder MI. Oral pathobiont induces systemic inflammation and metabolic changes associated with alteration of gut microbiota. Sci Rep. (2014) 4:4828. 10.1038/srep04828 24797416 PMC4010932

[24] Kato T Yamazaki K Nakajima M Date Y Kikuchi J Hase K. Oral administration of Porphyromonas gingivalis alters the gut microbiome and Serum metabolome. mSphere. (2018) 3(5):e00460-18. 10.1128/mSphere.00460-18 30333180 PMC6193602

[25] Sohn J Li L Zhang L Settem RP Honma K Sharma A. Porphyromonas gingivalis indirectly elicits intestinal inflammation by altering the gut microbiota and disrupting epithelial barrier function through IL9-producing CD4(+) T cells. Mol Oral Microbiol. (2022) 37(2):42–52. 10.1111/omi.12359 34958712 PMC9353576

[26] Li M Cui J Qu R Liu R Sun Y Li P. Porphyromonas gingivalis induces intestinal inflammation through gingipain-dependent gut microbiome dysbiosis. Microbiome. (2026) 14(1):148. 10.1186/s40168-026-02389-7 41923122 PMC13181988

[27] Pussinen PJ Könönen E Paju S Hyvärinen K Gursoy UK Huumonen S. Periodontal pathogen carriage, rather than periodontitis, determines the serum antibody levels. J Clin Periodontol. (2011) 38(5):405–11. 10.1111/j.1600-051X.2011.01703.x 21362013

[28] Massarenti L Nielsen CH Danielsen AK Jensen P Enevold C Damgaard C. Evaluation of circulating IgG antibodies against Porphyromonas gingivalis or its gingipains as serological markers of periodontitis and carriage of the bacterium. J Periodontol. (2025) 96(2):119–28. 10.1002/jper.23-0766 38884611 PMC11866731

[29] Papapanou PN Neiderud AM Disick E Lalla E Miller GC Dahlén G. Longitudinal stability of serum immunoglobulin G responses to periodontal bacteria. J Clin Periodontol. (2004) 31(11):985–90. 10.1111/j.1600-051X.2004.00599.x 15491314

[30] Slifka MK Antia R Whitmire JK Ahmed R. Humoral immunity due to long-lived plasma cells. Immunity. (1998) 8(3):363–72. 10.1016/s1074-7613(00)80541-5 9529153

[31] Murayama Y Nagai A Okamura K Kurihara H Nomura Y Kokeguchi S. Serum immunoglobulin G antibody to periodontal bacteria. Adv Dent Res. (1988) 2(2):339–45. 10.1177/08959374880020022401 3271028

[32] Hirai K Yamaguchi-Tomikawa T Eguchi T Maeda H Takashiba S. Identification and modification of Porphyromonas gingivalis cysteine protease, gingipain, ideal for screening periodontitis. Front Immunol. (2020) 11:1017. 10.3389/fimmu.2020.01017 32582160 PMC7290125

[33] Kudo C Naruishi K Maeda H Abiko Y Hino T Iwata M. Assessment of the plasma/serum IgG test to screen for periodontitis. J Dent Res. (2012) 91(12):1190–5. 10.1177/0022034512461796 23018816

[34] Joshi C Bapat R Anderson W Dawson D Cherukara G Hijazi K. Serum antibody response against periodontal bacteria and coronary heart disease: systematic review and meta-analysis. J Clin Periodontol. (2021) 48(12):1570–86. 10.1111/jcpe.13550 34545592

[35] Hoshino T Kaneko N Yoshihara A Iwasaki M Suwama K Ito Y. Associations between plasma antibody levels against Porphyromonas gingivalis and atrial fibrillation among community-dwelling older individuals in Japan: a cross-sectional study. Oral Health Prev Dent. (2023) 21:339–46. 10.3290/j.ohpd.b4528813 37861309 PMC11619824

[36] Bender P Bürgin WB Sculean A Eick S. Serum antibody levels against Porphyromonas gingivalis in patients with and without rheumatoid arthritis—a systematic review and meta-analysis. Clin Oral Investig. (2017) 21(1):33–42. 10.1007/s00784-016-1938-5 27561661

[37] Sparks Stein P Steffen MJ Smith C Jicha G Ebersole JL Abner E. Serum antibodies to periodontal pathogens are a risk factor for Alzheimer's Disease. Alzheimers Dement. (2012) 8(3):196–203. 10.1016/j.jalz.2011.04.006 22546352 PMC3712346

[38] Nakahara T Hyogo H Ono A Nagaoki Y Kawaoka T Miki D. Involvement of Porphyromonas gingivalis in the progression of non-alcoholic fatty liver disease. J Gastroenterol. (2018) 53(2):269–80. 10.1007/s00535-017-1368-4 28741270

[39] Iwauchi M Horigome A Ishikawa K Mikuni A Nakano M Xiao J. Relationship between oral and gut microbiota in elderly people. Immun Inflamm Dis. (2019) 7(3):229–36. 10.1002/iid3.266 31305026 PMC6688080

[40] Nakajima Y Kato-Kogoe N Yasuda T Urakawa R Matsuo T Omori M. Impact of periodontal treatment on early rheumatoid arthritis and the role of Porphyromonas gingivalis antibody titers. Med Sci Monit. (2025) 31:e947146. 10.12659/msm.947146 39863920 PMC11780957

[41] Nesse W Abbas F Van Der Ploeg I Spijkervet FK Dijkstra PU Vissink A. Periodontal inflamed surface area: quantifying inflammatory burden. J Clin Periodontol. (2008) 35(8):668–73. 10.1111/j.1600-051X.2008.01249.x 18564145

[42] Eke PI Page RC Wei L Thornton-Evans G Genco RJ. Update of the case definitions for population-based surveillance of periodontitis. J Periodontol. (2012) 83(12):1449–54. 10.1902/jop.2012.110664 22420873 PMC6005373

[43] Komori E Kato-Kogoe N Imai Y Sakaguchi S Taniguchi K Omori M. Gastrectomy-induced alterations in gut microbiota linked to changes in oral and gastric microbiota. Front Microbiol. (2025) 16:1599503. 10.3389/fmicb.2025.1599503 40606163 PMC12213597

[44] Omori M Kato-Kogoe N Sakaguchi S Fukui N Yamamoto K Nakajima Y. Comparative evaluation of microbial profiles of oral samples obtained at different collection time points and using different methods. Clin Oral Investig. (2021) 25(5):2779–89. 10.1007/s00784-020-03592-y 32975702

[45] Damgaard C Reinholdt J Enevold C Fiehn NE Nielsen CH Holmstrup P. Immunoglobulin G antibodies against Porphyromonas gingivalis or Aggregatibacter actinomycetemcomitans in cardiovascular disease and periodontitis. J Oral Microbiol. (2017) 9(1):1374154. 10.1080/20002297.2017.1374154 29081914 PMC5646634

[46] Lamont RJ Kuboniwa M. The polymicrobial pathogenicity of Porphyromonas gingivalis. Front Oral Health. (2024) 5:1404917. 10.3389/froh.2024.1404917 38736461 PMC11082793

[47] Sakanaka A Kuboniwa M Shimma S Alghamdi SA Mayumi S Lamont RJ. Fusobacterium nucleatum metabolically integrates commensals and pathogens in oral biofilms. mSystems. (2022) 7(4):e0017022. 10.1128/msystems.00170-22 35852319 PMC9426547

[48] Akase T Inubushi J Hayashi-Okada Y Shimizu Y. Association of Fusobacterium nucleatum in human saliva with periodontal status and composition of the salivary microbiome including periodontopathogens. Microbiol Spectr. (2024) 12(12):e0085524. 10.1128/spectrum.00855-24 39436120 PMC11619574

[49] Baima G Dabdoub S Thumbigere-Math V Ribaldone DG Caviglia GP Tenori L. Multi-omics signatures of periodontitis and periodontal therapy on the oral and gut microbiome. J Periodontal Res. (2025) 60(12):1237–53. 10.1111/jre.70055 41307322 PMC12881887

[50] Bao J Li L Zhang Y Wang M Chen F Ge S. Periodontitis may induce gut microbiota dysbiosis via salivary microbiota. Int J Oral Sci. (2022) 14(1):32. 10.1038/s41368-022-00183-3 35732628 PMC9217941

[51] Sterlin D Fieschi C Malphettes M Larsen M Gorochov G Fadlallah J. Immune/microbial interface perturbation in human IgA deficiency. Gut Microbes. (2019) 10(3):429–33. 10.1080/19490976.2018.1546520 30449244 PMC6546332

[52] Kato-Kogoe N Tsuda K Kudo A Sakaguchi S Omori M Komori E. Salivary microbiota and IgA responses are different in pre-diabetic individuals compared to normoglycemic controls. Front Cell Infect Microbiol. (2025) 15:1591285. 10.3389/fcimb.2025.1591285 40535541 PMC12174153

