# Supplementary material for: Serum antibody titers against Porphyromonas gingivalis are associated with oral and gut microbiota
Source: Front Oral Health. 2026 Sep 15;7:1956003. doi: 10.3389/froh.2026.1956003 (PMC13619529; doi:10.3389/froh.2026.1956003)
Supplement: Supplementary file 1 [file Image1.pdf]

Supplementary Material

Supplementary Figure

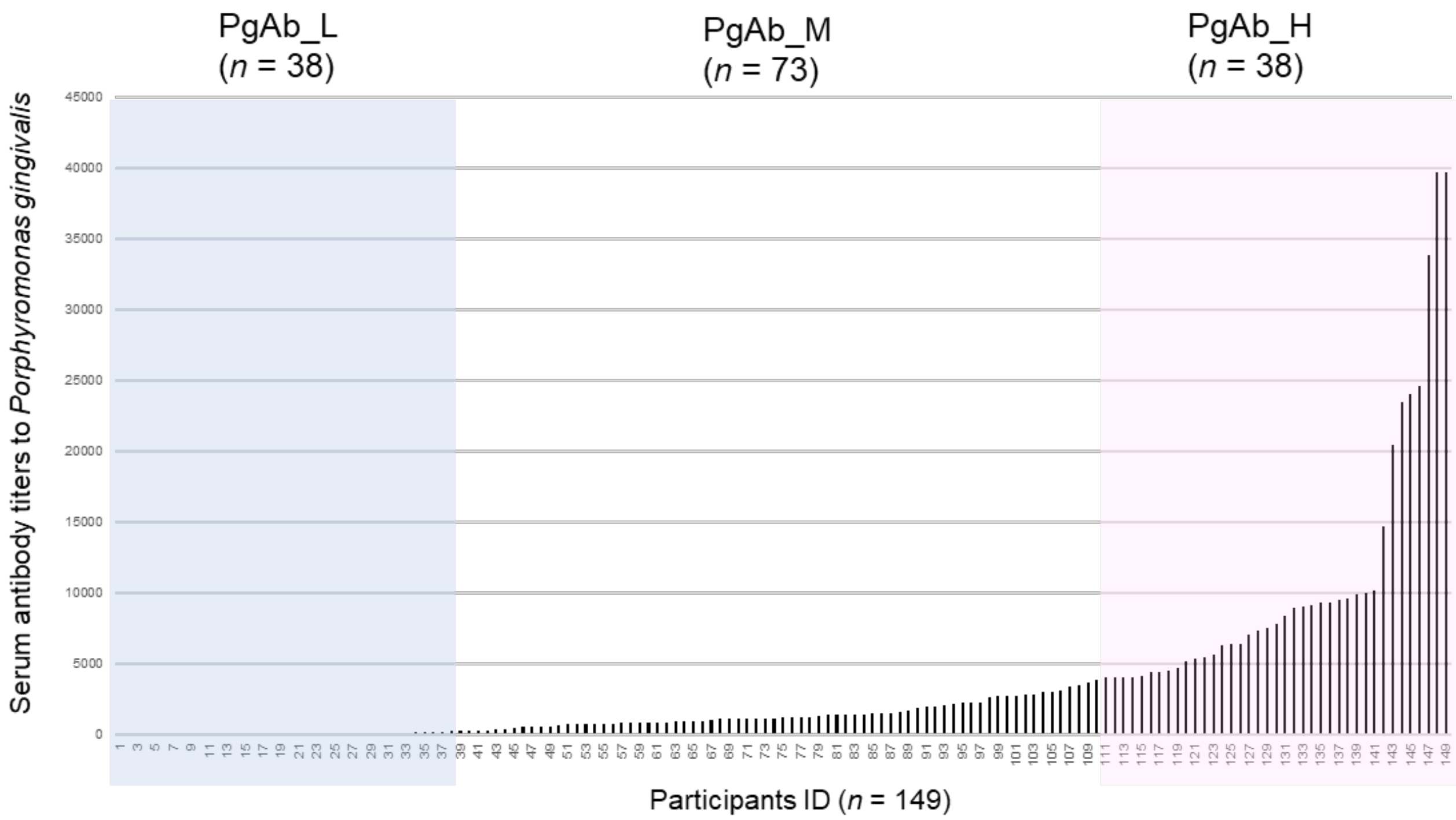

Supplementary Figure 1. The serum antibody titers against *P. gingivalis*

The serum antibody titers against *P. gingivalis* in each participant (*n* = 149) are shown. The participants were divided into three groups—high (PgAb_H), medium (PgAb_M), and low (PgAb_L)—based on the quartiles of their serum antibody titers against *P. gingivalis*.
